# Schools for Mothers

**Published:** 1914-08-29

**Authors:** Eric Pritchard, Eleanor Ritchie

**Affiliations:** Chairman of the Association of Infant Consultations and Schools for Mothers; Hon. Secretary of the Association of Infant Consultations and Schools for Mothers


### Schools for Mothers.

*To the Editor of* THE HOSPITAL.

SIR,—At this time of year the mortality among infants is always high, and since it is to the young generation that the country must eventually look for its strength, it is of the utmost importance at this juncture to prevent all unnecessary waste of life. At an emergency meeting of the Executive Committee of the Association of Infant Consultations and Schools for Mothers which has just been held, it was decided to ask the co-operation of the press to point out to all those who have the charge of young infants that many deaths and much illness may be prevented by the observance of a few quite simple principles of mothercraft, of which the most important are:

(1) To persevere with natural feeding up to the end of the ninth month, or longer, even though the mother is compelled to go out to work. Employers of female labour are earnestly requested to give nursing mothers opportunities for feeding their babies. (2) That in those cases in which such opportunities are impossible, weaning is by no means necessary. A supplementary feeding of cow's milk will not disagree with the infant, and may save the infant's life. (3) That the chief causes of illness among nursed and other babies is irregular and too frequent feeding by day as well as by night. Intervals by day should not be less than three hours, and at night a long interval of some six to eight hours should always be provided. (4) Expectant mothers should also seek advice as to their own health, either by consulting their doctors or by attending at schools for mothers. (5) That stimulants are not only unnecessary but are harmful, both to nursing and expectant mothers. —Yours, etc.,

ERIC PRITCHARD, Chairman of the Association of Infant Consultations and Schools for Mothers.

ELEANOR RITCHIE, Hon. Secretary of the Association of Infant Consultations and Schools for Mothers.

